# Supplementary material for: Polyketide synthase gene clusters in the ericoid mycorrhizal fungus Oidiodendron maius: genome-wide prediction, expression profiling, and functional characterization of knockout mutants
Source: Front Plant Sci. 2026 Sep 16;17:1924430. doi: 10.3389/fpls.2026.1924430 (PMC13623903; doi:10.3389/fpls.2026.1924430)
Supplement: Supplementary file 1 [file SupplementaryFile1.zip › Supplementary Materials/Supplementary Folder 1.zip!/Oidma1_AssemblyScaffolds_Repeatmasked/knownclusterblast/cluster13/jgi.p_Oidma1_157994_mibig_hits.html]

| MIBiG Protein | Description | MIBiG Cluster | MiBiG Product | % ID | % Coverage | BLAST Score | E-value |
| --- | --- | --- | --- | --- | --- | --- | --- |
| AAL06662.1 | epoxide\_hydrolase | BGC0000965 | C-1027\_biosynthetic\_gene\_cluster | 42 | 102.827763496 | 319 | 4.2e-87 |
| ALU98426.1 | epoxide\_hydrolase | BGC0001397 | Lidamycin\_biosynthetic\_gene\_cluster | 42 | 102.827763496 | 319 | 4.2e-87 |
| AFV52152.1 | epoxide\_hydrolase | BGC0000081 | Kedarcidin\_biosynthetic\_gene\_cluster | 42 | 99.2287917738 | 317 | 2.1e-86 |
| AAM78002.1 | epoxide\_hydrolase | BGC0000112 | Neocarzinostatin\_biosynthetic\_gene\_cluster | 41 | 100.514138817 | 313 | 4e-85 |
| AAM77995.1 | epoxide\_hydrolase | BGC0000112 | Neocarzinostatin\_biosynthetic\_gene\_cluster | 41 | 100.0 | 307 | 2.8e-83 |
| AFY23023.1 | hydrolase | BGC0000274 | Tetarimycin\_biosynthetic\_gene\_cluster | 40 | 101.028277635 | 305 | 1.1e-82 |
| ABP55155.1 | Epoxide\_hydrolase\_domain\_protein | BGC0000150 | Sporolide\_biosynthetic\_gene\_cluster | 41 | 99.4858611825 | 302 | 5.4e-82 |
| CAH60146.1 | IssA\_protein | BGC0000700 | Istamycin\_biosynthetic\_gene\_cluster | 39 | 100.514138817 | 270 | 3.8e-72 |
